# Changes in leg length and hip offset in navigated imageless vs. conventional total hip arthroplasty

**DOI:** 10.1038/s41598-023-44009-6

**Published:** 2023-10-11

**Authors:** Roberta Laggner, Anastasia Oktarina, Reinhard Windhager, Mathias P. G. Bostrom

**Affiliations:** 1 Department of Orthopedic Surgery, Hospital for Special Surgery, New York City, NY USA; 2 Department of Orthopedics and Trauma-Surgery, Medical University of Vienna, Waehringer Guertel 18-20, 1090 Vienna, Austria

**Keywords:** Bone, Orthopaedics

## Abstract

While previous studies on navigated total hip replacement (nTHA) focused on acetabular component positioning, we compared the results of nTHA with conventional total hip replacement (cTHA) in respect of changes in leg length and hip offset. In a single-center study results radiographic parameters of patients with unilateral THA were included. Data were retrospectively analyzed from computer navigation data and radiographs. Analysis concentrated on the discrepancy in leg length (LLD) and hip offset (OSD) between the affected and unaffected hip. The effect of the procedure was defined as the difference between postoperative and preoperative LLD and OSD values in each group. 2332 patients were analyzed. Both nTHA and cTHA were effective in restoring LLD and OSD by reducing the preoperative value significantly (p < 0.001). Regarding changes in LLD, no statistical difference between nTHA and cTHA could be found. Changes in OSD nTHA was a slightly more effective than cTHA (− 2.06 ± 6.00 mm vs. − 1.50 ± 5.35 mm; p < 0.05). Both navigated and conventional THA were successful in reconstruction of leg length and hip offset, while postoperative offset discrepancy was significantly lower in the navigated group at the cost of longer operation times. If these results are clinically relevant further investigation is needed.

## Introduction

Destruction of the hip joint cartilage caused by osteoarthritis leads to the alternation of the physiological center of rotation (COR) of the femoral head and contributes the impairment of hip joint function. Therefore, achieving proper biomechanics by reconstruction of the COR should be the aim of all total hip arthroplasties (THA)^1^.

Critical to restoring the COR is proper reconstruction of leg length and hip offset^2^. Leg length discrepancies (LLDs) after THA have been associated with unphysiological force transmission, hip instability, ipsilateral knee pain, sciatic nerve palsy, aseptic loosening, and early revision surgery. Besides resulting in unhappy patients, LLDs are often perceived by the patient and affects functional outcome^3^. Unphysiological postoperative LLDs are also a main cause for malpractice liability lawsuits after THA in the United^4^. Failure to restore hip offset postoperatively may lead to hip instability, impingement, and polyethylene wear^5,6^.

Hip offset comprises two components: acetabular offset and femoral offset. Acetabular offset affects hip stability, range of motion, and soft tissue tension, while femoral offset is dictated by the configuration of the stem and affects leg length and overall limb alignment^7,8^.

Regarding an optimal alignment of the acetabular component Lewinnek et al.^9^ recommended an inclination angle of 40° ± 10° and an anteversion angle of 15° ± 10° as a safe range for acetabular alignment in THA. Freehand techniques and the use of mechanical guides have been used in the past to ensure target values, but there has been a desire for even greater accuracy.

Imageless computer navigation in THA has emerged as a potential method of restoring limb length and acetabular offset with much greater accuracy than conventional instrumentation^10^. With the use of computer navigation, the cup position, femoral stem position, leg length and hip offset can be tracked intraoperatively with high precision. Consequently, computer navigation has been associated with a reduced rate of revision for dislocation following THA. Furthermore, in the component combinations most commonly used with navigation there was also a reduction in the rate of all-cause revision^1,11–13^.

Previous radiological studies mainly focused on the acetabular component and the precision of the measured anteversion and inclination, while the number of studies focusing on the leg length and hip offset reconstruction in imageless computer navigated total hip arthroplasty remains low^3,14–16^. Therefore, we compared the results of navigated (nTHA) with conventional total hip replacement (cTHA) in respect to leg length and hip offset reconstruction.

## Material and methods

In a retrospective single-center study collected data of patients with THA performed in 2019 and 2021 were evaluated and was approved by the institutional review board of our institution. Data were collected as a normal part of patients’ diagnosis and treatment as recorded in case notes, the departmental proprietary database, computer navigation data and radiographic images.

Two groups were reviewed, one group included all patients operated with navigated THA in 2021, and a control group of all patients operated with conventional THA operated in 2019. Due to the limited number of elective operations during the first lockdowns of CoV19-Pandemic patient data from 2020 could not be analyzed. A total of 3915 patients underwent nTHA or cTHA in the respective period of time. These operations were performed according to institutional standards; in the navigation group, surgery was performed using the Intellijoint HIP® imageless navigation device (IntellijointSurgical Inc., Kitchener, Ontario, Canada).

In these patients AP pelvic radiographs were performed before surgery and six weeks after in standard manner, with a marker of known diameter to account for magnification while postoperative radiographs were corrected for magnification using the acetabular liner diameter. The patients were supine position with x-ray beam centered over pubic symphysis with lower limbs placed in internal rotation with the big toes touching each other and patella facing forward. In this view the femoral neck could be viewed in full profile.

In the analysis only patients with unilateral hip disease were included. Excluded were patients with bilateral hip osteoarthritis, bilateral THA, inadequate pelvic radiographs, and revision surgeries as well as additional inserted implants such as cables and plates. All these patients had pre- and postoperative standard AP pelvic radiographs according to the institutional standard protocol.

**Figure 1:**
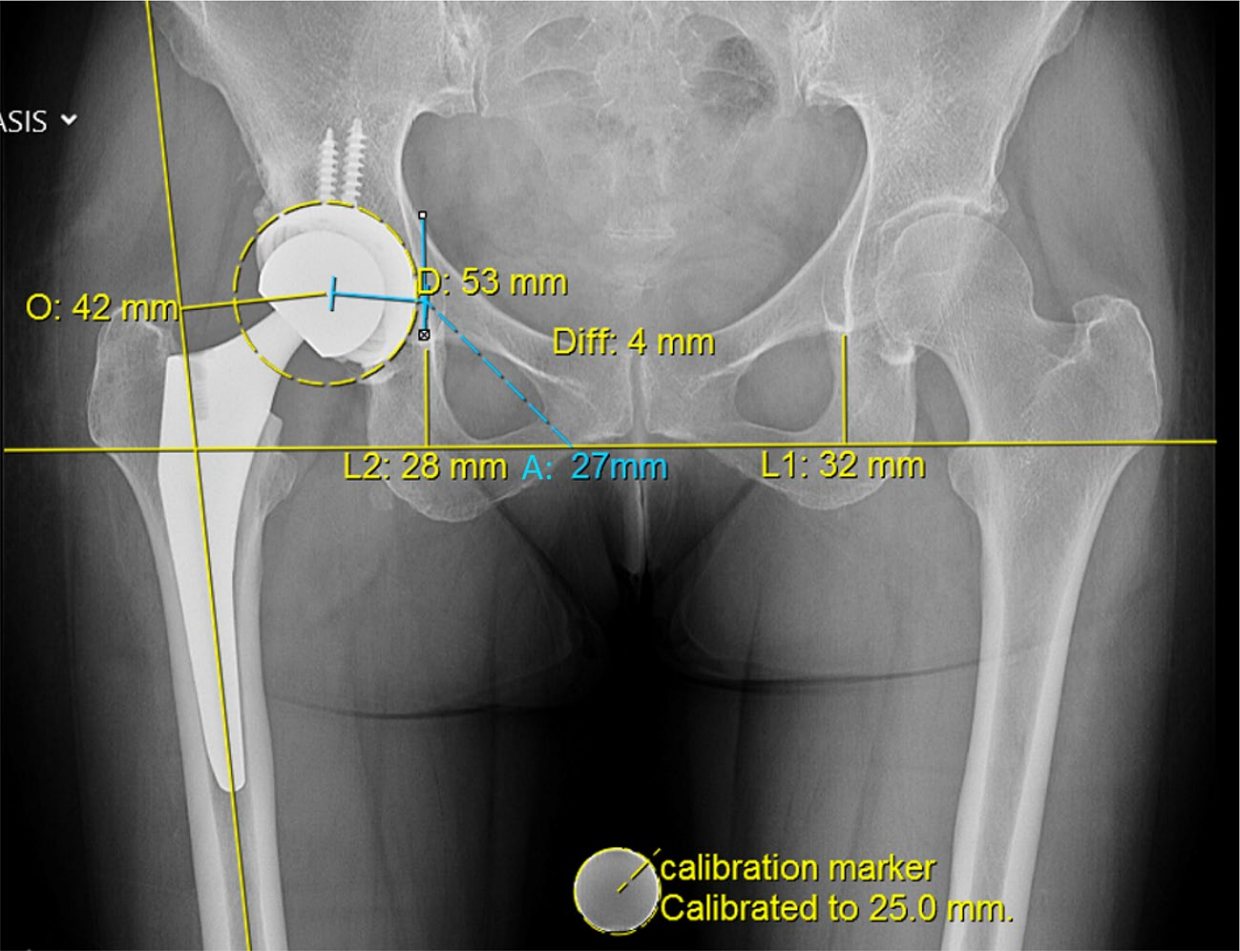
Leg length (LL) was measured with the Ranawat technique and hip offset (OS) was calculated as the sum of femoral and acetabular offset. Analysis of radiologic images concentrated on the discrepancy in leg length (LLD) and hip offset (OSD) between the affected and unaffected hip. (O: femoral offset, A: acetabular offset, Diff: LLD).

To calculate leg length (LL) the Ranawat technique was used^10,17,18^ (Fig. 1). The trans teardrop line was defined as the standard line. The perpendicular distance from this horizontal line to the apex of the lesser trochanter was measured and displayed in the Picture Archiving Communications System PACS templating system. The difference in between the affected und the contralateral unaffected hip was termed hip length discrepancy (HLD), which was determined in each patient pre- and postoperatively.

Hip offset (OS) was calculated as the sum of femoral and acetabular offsets^10^ Fig. 1). Femoral offset was defined as the perpendicular distance from the center of rotation of the femoral head to the line of action of the abductor muscles. Acetabular offset is the perpendicular distance from the center of rotation of the femoral head to the vertical trans-teardrop line. The difference in OS between the affected und the contralateral unaffected hip was termed offset discrepancy (OSD), which was determined in each patient pre- and postoperatively.

Radiographic cup anteversion was determined in shoot-through lateral radiographs by measuring the angle between the transverse axis and the acetabular opening^10,19^. For technical reasons shoot-through lateral radiographs were only taken in 861 patients. Therefore, anteversion was only determined in 454 patients with cTHA and 407 patients with nTHA.

All radiographs were accessed on Kodak PACS (Eastman Kodak Company, 10.1_SP1, 2006). All the radiographic measurements were performed retrospectively by a single author, who was not involved in patient care or surgery, using Orthoview Digital Planning Software system (Orthoview LLC) on PACS. To account inter-rater and intra-rater reliability 60 radiographs were randomly selected and the author (RL) measured them twice 2 weeks apart and they were also measured by another author independently (AO).

For statistical analysis continuous variables were reported as mean and standard deviation and categorial variables frequency and proportion. Cohorts were compared using Welch two sample t test for continuous data and Pearson Chi squared for categorical data. Levene's test using median as the center was used to compare variances between cohorts. Multivariable linear regression was conducted to investigate association of LLD and Offset discrepancy at postop with BMI and operative time. Significance was established at alpha 0.05.

The Institutional Review Board of the Hospital for Special Surgery approved the study protocol before data collection was started (IRB Number: 2022–0126).

All patients gave consent to treatment according to institutional guidelines and to anonymized assessment of clinical data and treatment outcome. This was a retrospective trial. Therefore, the Institutional Review Board waived the requirement to obtain distinct written informed consent from the patients.

### Ethics approval and consent to participate

The study was conducted according to the guidelines of the Declaration of Helsinki and was approved by the institutional review board of the Hospital for Special Surgery (IRB Number: 2022-0126).

### Informed consent

All patients gave consent to treatment according to institutional guidelines and to anonymized assessment of clinical data and treatment outcome. This was a retrospective trial. Therefore, the Institutional Review Board of the Hospital of Special Surgery waived the requirement to obtain distinct written informed consent from the patients.

## Results

Our final analysis included 2332 patients: 1174 patients with cTHA and 1158 patients with nTHA (Table 1). A total of 1583 patients were excluded due to the predefined exclusion criteria.

**Table 1:** Demographics of patients with conventional (cTHA) and navigated total hip arthroplasty (nTHA).

	cTHA (n = 1174)	nTHA (n = 1158)
Male (%)	523 (44.5)	577 (49.8)*
Age years (mean (SD))	63.0 (10.4)	61.7(9.8)**
BMI (mean (SD))	29.27 (6.00)	28.97 (5.59)
ASA (%)		
1	41 (3.5)	64 (5.5)*
2	951 (81.2)	951 ( 82.3)*
3–4	182 (15.3)	143 ( 12.2)*
Charlson Comorbidity Index1(%)		
0	829 (70.6)	847 (73.4)
1	164 (14.0)	172 (14.9)
2	127 (10.8)	97 (8.4)
3+	54 (4.6)	38 (3.3)

*p < 0.05.

**p < 0.001.

Due These two groups did not differ significantly in respect of BMI and Charlson Comorbidity index. However, in the nTHA group there were significantly more male patients than in the cTHA group (49.8% vs. 44.5; p = 0.012) and nTHA patients were also significantly younger (61.65 ± 9.79 vs. 63.04 ± 10.37; p = 000.9) Based on their ASA scores, patients of the nTHA appeared less medically compromised than patients with cTHA; ASA 3–4: cTHA n = 182 (15.3%) vs. nTHA143 (12.2%), p < 0.05.

Right sided THA operations were performed in 606 patients (52.3%) of nTHA group and in 643 patients (54.8%) of cTHA group (p = 0.255). Duration of operation in the nTHA group was significantly longer than in cTHA group (89.21 ± 24.63 min vs. 79.88 ± 23.29 min; p < 0.001).

Results of the analysis of the pelvic radiographs of the two groups are listed in Table 2. Both groups did not differ significantly in respect of pre- and postoperative LLD and preoperative OSD. Postoperative OSD was significantly lower in the nTHA group (− 0.92 ± 4.58 vs. − 0.07 ± 4.19; p < 0.0001). Inclination (40.47 ± 4.95 vs. 40.95 ± 5.28 degrees) and anteversion (19.41 ± 4.05 vs. 20.18 ± 4.95 degrees) of the acetabular cup were slightly but significantly (p < 0.05) lower in the nTHA group.

**Table 2:** Analysis of pelvic radiographs of patients with conventional (cTHA) and navigated total hip arthroplasty (nTHA).

	cTHA mean (sd) n = 1174	nTHA mean (sd) n = 1159
Leg length discrepancy preoperative (mm)	2.84 (3.10)	3.04 (3.31)
Leg length discrepancy postoperative (mm)	− 0.22 (2.83)	− 0.03 (2.77)
Effect of THA on leg length discrepancy (mm)	− 3.06 (3.62)*	− 3.07 (3.82)*^ns^
Hip offset discrepancy preoperative (mm)	1.44 (4.50)	1.11 (5.10)
Hip offset discrepancy postoperative (mm)	− 0.07 (4.19)	− 0.95 (4.58)^#^
Effect of THA on hip offset discrepancy (mm)	− 1.51 (5.35)*	− 2.06 (6.00)*^#^
Inclination postoperative (degrees)	40.95 (5.28)	40.47 (4.95)#
Anteversion^§^ postoperative (degrees)	20.18 (4.95)	19.41 (4.05)#

^§^For technical reasons anteversion was only determined in 454 patients with cTHA and 407 patients with nTHA.

*p < 0.001 postoperative vs. preoperative.

^ns^cTHA vs. nTHA.

^#^p < 0.05 cTHA vs. nTHA.

Both nTHA and cTHA were effective in restoring LLD and OSD by reducing the preoperative value significantly (p < 0.001). Regarding changes in LLD no statistical difference between nTHA and cTHA could be found. Regarding OSD, nTHA was slightly more effective than cTHA (− 2.06 ± 6.00 vs. − 1.50 ± 5.35; p < 0.05).

Testing for variance revealed no significant differences between the two groups for LLD post-operative (F = 2.6186, p = 0.1058), offset discrepancy postoperative (F = 0.4785, p = 0.4892) and inclination (F = 2.0801, p = 0.1494). Anteversion in cTHA had a significantly higher variance than in nTHA (F = 13.452, p < 0.001).

Linear regression analysis was performed to study factors associated with LLD postoperatively (Table 3). Navigated Operation (p = 0.186) and operative time (p = 0.103) had no influence, BMI showed a trending significant association with LLD postoperatively (p = 0.070). Linear regression analysis was also performed to study factors associated with OSD postoperatively (Table 4). BMI showed no significant association (p = 0.142), while significant associations were found for nTHA (p < 0.001) and operative time (p = 0.006).

**Table 3:** Linear regression analysis of factors associated with postoperative LLD.

	Estimate	Std. Error	p value
Navigated (ref = conventional)	0.57	0.119	0.186
BMI	0.018	0.010	0.070*
Operative time	0.004	0.002	0.103

*Trending significance, BMI is associated with LLD.

**Table 4:** Linear regression analysis of factors associated with postoperative OSD.

	Estimate	Std. Error	p value
Navigated * (ref = conventional)	− 0.944	0.194	< 0.001
BMI	− 0.024	0.017	0.142
OperativeTime#	0.011	0.004	0.006

*nTHA is associated with lower postoperative OSD than cTHA.

^#^Operative time is associated with postoperative OSD.

## Discussion

We retrospectively analyzed radiographs of patients who underwent cTHA in 2019 and nTHA 2021 in our institution. We are aware of the fact that no randomization was performed. Patients with cTHA were older, more often female and had higher ASA scores than patients with nTHA. All these limitations must be considered, when the two groups are compared.

These limitations do not however compromise the finding of this study that navigated hip arthroplasties need longer operation times with a mean prolongation of 10 min when navigation is used. This has been well documented by others^1,20–24^. While newer observations suggest that with a reasonable learning curve and improved workflow no added operative time in imageless navigation THA can be achieved^25^, we found no such change with increased use of navigation. In addition to the longer operative times, the higher costs of the navigated operation must be considered^1^ but was not the focus of this study.

We found that in both groups the operations were successful in restoring leg length and hip offset discrepancy. While postoperative LLD was not associated with the mode of operation or operative time, there was a trend of increased LLD with increased BMI. Computed tomography measurements have shown that measurements of inclination and anteversion were affected by the thickness of the soft tissue overlying the anterior superior iliac spine and pubic tubercles^26^. It is reasonable that our observation is due to different thicknesses of soft tissue. Interestingly, in contrast to published data, we could not document significantly higher decreases in leg length discrepancies postoperatively with computer navigation versus conventional THA^21,24^. Our results show, that in restoring hip offset discrepancy nTHA was statistically more effective than cTHA as it was associated with lower OSD values. Whether this discrepancy difference which was less than 1 mm is clinically relevant is unclear.

Navigated THA is advocated for improved placement of the acetabular component within the Lewinnek safe zone defined by a cup inclination of 40° ± 10° and a cup anteversion of 15° ± 10°^9,20,27–32^ Although cup placement within this zone has not been shown to significantly reduce dislocation rates, it is still a clinically relevant measure (32, 33). Regarding acetabular cup inclination and anteversion our results show that both surgical approaches resulted in acetabular positions which are well within Lewinnek safe zone^9,33^. The slightly but significant lower values in the nTHA group are noteworthy. The significantly higher variance of anteversion in cTHA is implicates that statistically nTHA is more precise than cTHA. Also, recent research demonstrates in vivo that an imageless, non-invasive navigation system is a reliable tool for intra-operative leg length and offset when compared to standard practice of plain film radiographs^34^.

Our study has several limitations. It is a retrospective, non-randomized analysis of groups of patients, that were treated in different time periods, with different implants. These groups differed statistically in respect of age, sex, and ASA-Scores. Therefore, all radiological outcome parameters must be interpreted with caution. We can only speculate that the radiologically observed differences between the two groups lead to clinically relevant differences in the follow up. However, sparce data on long term results imply that these differences do not achieve clinical relevance^30^.

In summary our results on the one hand confirm published data that the navigated approach to total hip arthroplasty offers higher precision in cup placement and off set at the cost of longer operation times, while, on the other hand, we were able to demonstrate no radiographically superiority of navigation with regard to leg length reconstruction.

If these results are clinically relevant or affect long term clinical outcomes, needs further investigations.

## Data Availability

The datasets used and/or analyzed during the current study are available from the corresponding author on reasonable request.
